# Case Report: A rare case of over 45 years’ survival in a patient with tonsillar adenoid cystic carcinoma

**DOI:** 10.3389/fonc.2026.1824507

**Published:** 2026-06-02

**Authors:** Mingzhu Wang, Tingyao Ma, Yue Zhao, Shujing Zhang, Xuelian Wang, Guoliang Yang, Xi Zhao, Yixuan Liu, Tian Ye, Mengmeng Li, Jun Wu, Lanlan Xuan, Xiaohong Chen, Zheng Yang

**Affiliations:** 1 Department of Thyroid and Head and Neck Surgery, Beijing Tongren Hospital, Capital Medical University, Beijing, China; 2 Department of Pathology, Anqing Hospital Affiliated to China Pharmaceutical University/Anqing Municipal Hospital, Anqing, Anhui, China

**Keywords:** adenoid cystic carcinoma, genetic analysis, long-term survival, recurrent head and neck tumor, tonsillar adenoid cystic carcinoma

## Abstract

Tonsillar adenoid cystic carcinoma (TACC) is an extremely rare malignancy with an unpredictable clinical course. We report an unusual case of TACC with a 47-year disease trajectory, characterized by prolonged indolent behavior followed by late and relatively rapid clinical progression. The patient was initially diagnosed with left tonsillar ACC in 1977 after presenting with dysphagia and underwent tonsillectomy followed by postoperative radiotherapy. A local recurrence involving the left soft palate occurred in 1996–1997 and was treated with surgical resection and tongue flap reconstruction, after which the disease remained clinically stable for many years. In 2018, recurrent ACC of the soft palate was confirmed in the radical resection specimen, and the patient underwent radical resection via a mandibulotomy approach followed by reconstruction and adjuvant concurrent chemoradiotherapy. Molecular profiling of the available 2018 recurrent soft palate lesion revealed NOTCH2 copy number gain and structural variants involving MTOR and SPEN. Regional cervical lymph node metastasis was confirmed in 2022, followed by intracranial disease progression in 2023. The patient died in 2024 from complications of progressive intracranial and systemic metastases. To our knowledge, this represents one of the longest documented clinical courses of TACC with integrated genomic analysis. This case highlights the potential for TACC to remain indolent for decades before transitioning to late regional and systemic progression, underscoring the importance of long-term surveillance and comprehensive clinicopathological and molecular evaluation.

## Introduction

Adenoid cystic carcinoma (ACC) is a rare malignant tumor that commonly arises in the salivary glands and is characterized by slow but persistent progression, perineural invasion, and a high rate of distant metastasis (1). While it most frequently originates in the major salivary glands, primary ACC of the oropharyngeal region, such as the palatine tonsil, is exceedingly uncommon. Even rarer are cases demonstrating late-onset distant or locoregional metastases after decades of apparent clinical quiescence.

The molecular pathogenesis of ACC is complex and incompletely understood. Classic alterations involve MYB-NFIB fusions (2), but recent studies have identified Notch pathway aberrations, mTOR activation, and epigenetic regulators as additional contributors to tumor behavior and progression (3). NOTCH2 amplification has been associated with aggressive phenotypes in various tumor types, whereas SPEN mutations may cause transcriptional repression within the Notch pathway, further enhancing oncogenic signaling.

Here, we present a unique case of a patient with primary TACC with soft palate metastasis, who displayed an unusually long indolent phase of more than 40 years, followed by rapid disease progression in the final 6 years. Molecular profiling of the metastatic lesion revealed NOTCH2 copy number gain without classical NOTCH mutations, accompanied by structural variants in SPEN and mTOR, suggesting a potential mechanism for late-stage transformation. This case offers novel insights into the dynamic molecular evolution of ACC and highlights the role of genomic instability in mediating the transition from indolent to aggressive disease states.

## Case presentation

A 58-year-old Han Chinese woman presented to Beijing Tongren Hospital in 2018 with a 6-month history of progressive otalgia and pruritus. She had a history of hypertension, no history of tobacco or alcohol use, and no known family history of malignancy.

### Initial diagnosis and long-term local disease course

Her previous oncologic history was notable for left tonsillar adenoid cystic carcinoma, initially diagnosed in 1977, followed by a remarkably prolonged clinical course. Because of the exceptionally long disease course, a concise chronological timeline of the major clinical milestones is provided in **Table 1**.

**Table 1:** Chronological timeline of the patient’s 47-year disease course.

Year/date	Disease phase	Clinical milestone	Management/known treatment details	Outcome/notes
1977	Primary local disease	Primary left tonsillar ACC diagnosed after biopsy for dysphagia	Left tonsillectomy followed by postoperative radiotherapy	Detailed radiotherapy records, including dose, fractionation, and treatment duration, were unavailable
1996–1997	Local recurrence	Lesion involving the left soft palate identified after odynophagia	Left soft palate resection with tongue flap reconstruction in January 1997	Interpreted as local recurrence rather than metastasis because the lesion was adjacent to the original tonsillar site and no nodal or distant disease was documented at that time; long clinically stable interval followed
1997–early 2018	Long clinically stable interval	No documented nodal or distant progression during long-term follow-up	Regular follow-up; no evidence-based systemic anticancer therapy documented	Prolonged clinically stable interval before suspected local recurrence in 2018
Early 2018, first admission	Suspected local recurrence	Progressive otalgia and pruritus; imaging showed a left tonsillar fossa/soft palate lesion	Initial diagnostic local excision was performed to distinguish post-treatment fibrosis/inflammation from tumor recurrence	Frozen-section assessment and H&E review of limited diagnostic tissue showed focal carcinoma infiltration suspicious for recurrent ACC; permanent sections of additionally submitted local excision/margin tissue showed chronic inflammation without definite residual carcinoma; definitive tumor classification was deferred
2018-02-08, second admission	Confirmed local recurrence	Recurrent left soft palate ACC confirmed in the radical resection specimen	Radical resection via mandibulotomy with temporalis muscle flap reconstruction and abdominal fat grafting	Pathology confirmed recurrent ACC with infiltrative growth, focal perineural invasion, cauterized surface involvement, and Ki-67 approximately 30%
2018 postoperative period	Adjuvant treatment and molecular profiling	Adverse pathological features prompted adjuvant treatment; molecular profiling was performed on the available 2018 recurrent soft palate lesion	Postoperative concurrent chemoradiotherapy was administered; radiotherapy was delivered as 60 Gy using VMAT; exact concurrent chemotherapy regimen, dosage, and number of cycles could not be fully retrieved	Treatment was completed as planned; NGS revealed NOTCH2 copy number gain and structural variants involving MTOR and SPEN
2020	Suspected regional cervical nodal metastasis	Right cervical mass detected	Close observation was initially recommended because of the indolent course and absence of symptoms	Clinically suspected regional cervical nodal metastasis
2022	Confirmed regional cervical nodal metastasis	Cervical MRI showed cervical lymphadenopathy and abnormal enhancement involving the left parotid/skull base region; FNA of a right level II lymph node confirmed metastatic ACC	Bilateral neck dissection, tracheotomy, and biopsy of a left upper alveolar gingival lesion were performed	Multiple cervical nodal metastases were confirmed; the gingival specimen showed chronic inflammatory changes only
June 2023	Surveillance/systemic assessment	Previously noted pulmonary nodule remained stable	Continued surveillance	No definite pulmonary progression documented
November 2023	Distant progression	Intracranial metastasis detected at an outside institution	Further disease-directed therapy was not pursued after discussion with the patient and family	Transition to supportive and palliative care
2024-03-11	Supportive/palliative care	Progressive intracranial and systemic disease	Symptom-directed supportive and palliative management	The patient died from complications of progressive intracranial and systemic metastases

Available treatment details are summarized according to the retrievable medical records. Detailed records of the 1977 postoperative radiotherapy, including dose, fractionation, and treatment duration, were unavailable because of the long interval since treatment. For the 2018 postoperative treatment, radiotherapy was delivered as 60 Gy using volumetric modulated arc therapy; however, the exact concurrent chemotherapy regimen, dosage, and number of cycles could not be fully retrieved.

ACC, adenoid cystic carcinoma; FNA, fine-needle aspiration; NGS, next-generation sequencing; VMAT, volumetric modulated arc therapy.

In 1977, at 18 years of age, the patient presented with dysphagia and was diagnosed with ACC of the left tonsil after biopsy. She underwent left tonsillectomy followed by postoperative radiotherapy, although detailed treatment records from that period were unavailable. In 1996, she developed odynophagia, and a lesion involving the left soft palate was identified. Given its proximity to the original tonsillar site and the absence of documented nodal or distant disease at that time, the lesion was interpreted as a local recurrence rather than a metastatic focus. Surgical resection with tongue flap reconstruction was performed in January 1997, after which she remained clinically stable for many years.

### 2018 local recurrence, pathological confirmation, and treatment

**Figure 1:**
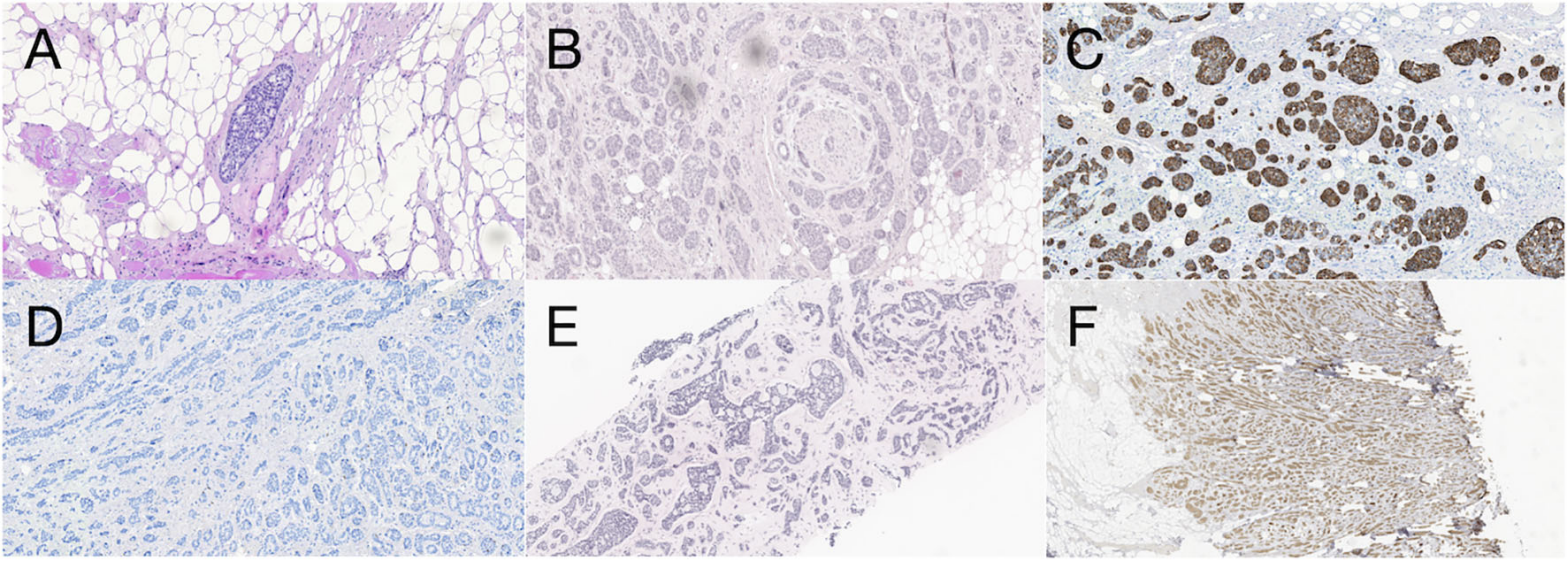
Histopathological confirmation and differential diagnostic workup of recurrent and metastatic adenoid cystic carcinoma. **(A)** H&E-stained small diagnostic tissue fragment with fibroadipose/stromal tissue and a focal infiltrative basaloid tumour area. **(B)** 2018 resection specimen showing infiltrative tumour nests with tubular, cribriform, and focal solid patterns in hyalinized stroma. **(C)** p40 immunostaining shows no diffuse nuclear tumour positivity. **(D)** CK5/6 immunostaining shows partial tumour-cell positivity. **(E)** 2022 cervical lymph node biopsy showing metastatic tumour nests with cribriform and tubular architecture. **(F)** Ki-67 immunostaining shows scattered brown nuclear positivity, with a proliferation index of approximately 10%.

At presentation in 2018, physical examination showed postoperative changes after prior tonsillectomy and soft palate reconstruction, with an irregular, white-coated lesion on the reconstructed left soft palate. Contrast-enhanced MRI and CT demonstrated a soft tissue lesion in the left tonsillar fossa/soft palate region without definite adjacent bone invasion (**Supplementary Figures 1A, B**). Because postoperative distortion and mucosal irregularity made clinical assessment difficult, and imaging could not reliably distinguish post-treatment fibrosis, chronic inflammation, and tumor recurrence, diagnostic excision was performed during the first 2018 admission. Intraoperative frozen-section assessment of the initial diagnostic local excision showed focal carcinoma infiltration suspicious for recurrent ACC in the clinical context (**Figure 1A**). However, the final permanent-section report of the additionally submitted local excision showed chronic inflammatory changes without definite residual carcinoma. Therefore, the initial diagnostic excision was regarded as evidence raising suspicion for recurrent carcinoma, but not as the sole basis for definitive tumor classification.

During the second 2018 admission, the patient underwent radical resection of the left soft palate lesion via a mandibulotomy approach on February 8, 2018, followed by temporalis muscle flap reconstruction and abdominal fat grafting. Final pathological examination of the resection specimen confirmed recurrent ACC measuring approximately 2.0 × 2.0 × 0.8cm, with infiltrative growth and focal perineural invasion (**Figure 1B**). Tumor involvement was noted at the cauterized resection surface, while lymphovascular invasion could not be definitively assessed based on the available pathological material. Immunohistochemical analysis showed positivity for CK7, CK8/18, and CD117, partial expression of p63, and a Ki-67 proliferation index of approximately 30%. Detailed pathological findings and immunohistochemical results from the 2018 diagnostic local excision, the 2018 radical resection specimen, and the 2022 metastatic cervical lymph node specimens are summarized in **Supplementary Table 1**.

Non-keratinizing squamous cell carcinoma was specifically considered in the differential diagnosis because of the oropharyngeal location and potential basaloid morphological overlap. Additional p40 and CK5/6 staining was performed on the 2018 radical resection specimen. p40 was negative in the tumor cells, and CK5/6 showed only partial positivity rather than diffuse sheet-like squamous marker expression (**Figures 1C, D**). Together with the absence of overt squamous differentiation, characteristic ACC-like cribriform/tubular architecture, pseudocystic basement membrane-like material, focal perineural invasion, strong CD117 expression, and the patient’s prior history of tonsillar ACC, these findings supported recurrent ACC rather than non-keratinizing squamous cell carcinoma. Other salivary gland-type tumors, including mucoepidermoid carcinoma, polymorphous adenocarcinoma, basal cell adenocarcinoma, and pleomorphic adenoma, were excluded based on morphology, immunophenotype, infiltrative growth pattern, and clinical history.

Given the adverse pathological features, postoperative concurrent chemoradiotherapy was administered and completed as planned. Available and unavailable treatment details are summarized in **Table 1**.

Molecular profiling of the available 2018 recurrent soft palate lesion revealed NOTCH2 copy number gain (**Supplementary Table 2**) and structural variants involving MTOR and SPEN (**Supplementary Table 3**). The sequencing data were deposited in the Genome Sequence Archive for Human under accession number HRA07116.

### Regional cervical nodal metastasis, distant progression, and outcome

In 2020, a right cervical mass was detected and clinically suspected to represent regional cervical nodal metastasis (**Supplementary Figure 2A**).

In 2022, neck MRI showed cervical lymphadenopathy and abnormal enhancement involving the skull base, left cavernous sinus, sphenoid sinus region, and left upper alveolar region, suggesting regional disease progression or locoregional extension (**Supplementary Figures 2B–D**). Ultrasound-guided fine-needle aspiration of a right level II lymph node confirmed metastatic ACC (**Figures 1E, F**). he patient subsequently underwent bilateral neck dissection, tracheotomy, and biopsy of a left upper alveolar gingival lesion. Metastatic ACC was identified in multiple cervical lymph nodes, whereas the gingival specimen showed chronic inflammatory changes only. The metastatic lymph nodes showed predominantly cribriform architecture with a tubular component and a Ki-67 index of approximately 10%.

These findings indicated a transition from a prolonged indolent disease course to late and relatively rapid clinical progression. The pulmonary nodule remained radiologically stable on serial chest CT during follow-up (**Supplementary Figure 3**); however, brain metastasis was detected at an outside institution in November 2023. After discussion with the patient and her family, no further disease-directed therapy was pursued because of advanced systemic progression and limited expected benefit. The patient received supportive and palliative care and died on March 11, 2024, due to complications of progressive intracranial and systemic metastases.

## Discussion

ACC is a rare malignant epithelial tumor that most commonly arises from the minor salivary glands and accounts for approximately 1% of all head and neck malignancies (4). It is characterized by indolent growth, a marked tendency for perineural invasion, and early hematogenous dissemination (5), whereas regional lymph node metastasis is relatively uncommon, occurring in fewer than 10% of cases (6). Despite its apparently slow growth kinetics, ACC is well recognized for its high incidence of delayed local recurrence and distant metastasis, which ultimately compromises long-term survival.

Primary TACC is exceptionally rare, with only a small number of cases reported in the literature. In our targeted literature review, fewer than 10 eligible cases of primary tonsillar ACC were identified. For contextual comparison, we also reviewed selected ACCs arising from anatomically related minor salivary gland sites of the oropharynx, while excluding tumors originating from major salivary glands, paranasal sinuses, or lacrimal glands. **Table 2** summarizes patient demographics, tumor features, treatment, outcomes, and, where available, molecular findings of primary tonsillar ACC cases and selected oropharyngeal minor salivary gland ACCs included for contextual comparison. Our case is notable for its prolonged clinical course, including repeated local recurrence, delayed regional nodal disease, distant progression, and molecular profiling of the available recurrent lesion. These features distinguish it from previously reported cases, which have relied mainly on histopathology and short-term follow-up. Long-term survival exceeding two decades appears to be exceedingly uncommon. A targeted PubMed search was performed from inception to December 2025 using predefined keywords; detailed search strategy, inclusion, and exclusion criteria are provided in **Supplementary Table 4**. The literature search and selection process are summarized in **Supplementary Figure 4** using a PRISMA-style flowchart.

**Table 2:** Reported cases of primary tonsillar ACC and selected oropharyngeal minor salivary gland ACCs used for contextual comparison.

No.	Author (year)	Country	Age (years)/ sex	Case category	Primary site	Histological pattern	Treatment	Recurrence	Metastasis/disease extension*	Follow-up duration	Outcome	Molecular/genomic data	Reference
1	Guo et al. (2024)	China	50/F	Primary tonsillar ACC	Left palatine tonsil (upper pole)	Cribriform	Surgery + postoperative RT 60 Gy; END not performed	No	NR	12 months	NED	Not reported	(17)
2	Azizli et al. (2011)	Turkey	49/F	Primary tonsillar ACC	Bilateral palatine tonsils (left parenchyma + right peritonsillar tissue)	Mixed (cribriform predominance + tubular)	Bilateral tonsillectomy + postoperative RT	No	No	12 months	NED	No genomic testing reported	(18)
3	Pushpanjali et al. (2014)	India	50/F	Contextual oropharyngeal minor salivary gland ACC	Right oropharynx (soft palate to palatine tonsil)	Cribriform (focal solid)	Surgical excision + postoperative RT	No	No	18 months	NED	No genomic testing reported	(19)
4	Schwartz et al. (2019)	USA	70/F	Contextual oropharyngeal minor salivary gland ACC	Suspected soft palate/oropharyngeal minor salivary gland source	Mixed cribriform and tubular	IMRT 54 Gy to retro-orbital areas and skull base; SRS for stable L2 vertebral lesion	NR	Possible L2 vertebral lesion; bilateral orbital/skull base involvement via perineural extension	27 months	AWD; radiographic PR/SD	Not reported	(20)
5	Kobayashi et al. (2021)	Japan	76/M	Contextual oropharyngeal minor salivary gland ACC	Right oropharynx	ACC; histological pattern NR	C-ion RT (primary) + SBRT (lung metastases)	NR	Yes (lung)	21 months	AWD; local control achieved after RT	Not reported	(21)
6	Bouchlarhem et al. (2025)	Morocco	42/M	Contextual oropharyngeal minor salivary gland ACC	Hard–soft palate (minor salivary gland)	Solid subtype (Szanto grade III), PNI+	Surgery + adjuvant IMRT (66 Gy)	No	No	12 months	NED	No genomic testing reported	(22)
7	Present case	China	58/F	Primary tonsillar ACC	Left palatine tonsil	2018: Infiltrative ACC with focal perineural invasion; 2022 LN: Cribriform (80%) + tubular (20%)	1977: tonsillectomy + postoperative RT, details NR; 1997: local resection + tongue flap reconstruction; 2018: radical resection + adjuvant CCRT; 2022: bilateral neck dissection	Yes (1996–1997 and 2018 local recurrence involving the left soft palate)	Yes (regional cervical LNs, 2020–2022; intracranial metastasis, 2023)	564 months (47 years)	DOD	NOTCH2 copy number gain; structural variants involving MTOR and SPEN	Present study

Primary tonsillar ACC cases and selected non-tonsillar oropharyngeal minor salivary gland ACC cases are categorized separately. Non-tonsillar cases were included only for contextual comparison and were not counted as primary TACC.

*Disease extension includes perineural skull base/orbital extension when reported.

ACC, adenoid cystic carcinoma; AWD, alive with disease; CCRT, concurrent chemoradiotherapy; C-ion RT, carbon-ion radiotherapy; DOD, died of disease; END, elective neck dissection; IMRT, intensity-modulated radiotherapy; LN, lymph node; NED, no evidence of disease; NR, not reported; PNI, perineural invasion; PR, partial response; RT, radiotherapy; SBRT, stereotactic body radiotherapy; SD, stable disease; VMAT, volumetric modulated arc therapy.

Based on available cohort studies of malignant minor salivary gland tumors of the oropharynx, the reported 5-year and 10-year overall survival rates are approximately 80% and 53%, respectively, and the recurrence-free survival rates range from 60% to 70% (7). Nevertheless, nearly 60% of patients with ACC ultimately develop recurrence after initial treatment, and the lung remains the most frequent site of distant metastasis. The 5-year local control rate is reported at 69%, while the 10-year distant metastasis rate ranges from 20% to 55% (8). Histologically, compared with the solid subtype, cribriform and tubular growth patterns are generally associated with more favorable outcomes. However, given the tumor’s prolonged natural history and propensity for late relapse, long-term—often lifelong—surveillance is widely recommended, and sustained disease control rather than a definitive cure is increasingly regarded as a realistic therapeutic objective for patients with ACC.

The present case provides a rare opportunity to observe the long-term natural history of TACC, characterized by an exceptionally long period of relative clinical stability followed by late and relatively rapid clinical progression. This biphasic disease trajectory, although increasingly recognized as a hallmark of ACC, has been only sporadically documented, particularly in tumors arising from the palatine tonsil.

ACCs are well known for their indolent growth patterns and their capacity for prolonged clinical quiescence, during which residual or disseminated tumor cells may remain clinically undetectable for many years before reactivation. Previous reports have described extremely delayed recurrence or distant metastasis occurring more than one to two decades after initial treatment, underscoring the distinctive natural history of this malignancy. For example, one reported case described metastatic ACC identified 19 years after primary tumor resection, highlighting the potential for very delayed progression after prolonged clinical quiescence (9).

Several clinicopathological observations may help contextualize such an extended survival interval. From a histopathological perspective, ACCs with predominantly cribriform or tubular growth patterns are generally associated with lower proliferative activity and slower clinical evolution, which may facilitate long-term disease control. In addition, emerging molecular studies have proposed biologically distinct subtypes of ACCs, including MYB-driven tumors that tend to exhibit relatively indolent behavior and NOTCH-altered subgroups associated with more aggressive progression. Because molecular profiling was not available for the early lesions, the biological basis of the prolonged stable interval cannot be determined. The cribriform/tubular morphology and relatively low proliferative features observed in later specimens may provide clinicopathological context, but they cannot establish the molecular state of the original tumor or explain the subsequent acceleration.

During this prolonged course, the disease remained predominantly locoregional for decades before cervical nodal involvement and subsequent distant progression were documented. Although the biological basis for this transition cannot be determined from a single case, previous studies have suggested that lymph node metastasis in ACC may be associated with an increased risk of distant dissemination, particularly when multiple nodes are involved (10, 11). This observation is consistent with the clinical acceleration seen in the later phase of the present case, but it does not establish a causal relationship.

Recurrent or metastatic ACCs have been reported to show greater genomic complexity and intertumoral heterogeneity than primary lesions (12, 14). In the present case, molecular profiling was performed on the available 2018 recurrent soft palate lesion because earlier archival specimens were unavailable. The analysis revealed NOTCH2 copy number gain and structural variants involving SPEN and MTOR. These findings are noteworthy because NOTCH pathway alterations and broader genomic complexity have been reported in aggressive subsets of ACC (12–14). However, they should be interpreted with caution. Because archival tumor specimens from the 1977 primary lesion and the 1997 soft palate recurrence were unavailable, longitudinal genomic comparison could not be performed. In addition, no functional validation was conducted. Therefore, the observed NOTCH2, SPEN, and MTOR alterations cannot establish a causal mechanism for late disease acceleration. Rather, they should be regarded as hypothesis-generating molecular findings that may reflect genomic complexity acquired or selected during the long clinical course. Further studies with paired primary, recurrent, and metastatic samples are needed to determine whether similar alterations contribute to clonal evolution, tumor dormancy, or late progression in TACC (12).

Collectively, this case illustrates that prolonged survival in ACC does not necessarily indicate definitive biological cure, because clinically indolent disease may still be followed by late regional or distant progression (15, 16). However, the mechanisms underlying this transition cannot be determined from this single retrospective case. The observed clinical course and molecular findings from the available 2018 recurrent lesion should therefore be interpreted as hypothesis-generating observations. This case reinforces the importance of lifelong surveillance in patients with ACC and highlights the need for future studies using paired longitudinal specimens to clarify the biological basis of prolonged clinical quiescence and late progression (12, 15, 16).

Several important limitations should be acknowledged. First, clinical photographs of the primary tonsillar lesion and early disease course were unavailable because of the retrospective nature of this case and the long interval since the initial diagnosis and treatment. This limits the visual documentation, educational value, and overall completeness of this rare case report. Second, original pathological material from the 1977 primary tonsillar lesion and the 1997 soft palate recurrence was not available for central pathological review, additional immunohistochemical staining, or molecular testing. As a result, direct histopathological and genomic comparison between the primary tumor, early recurrence, late recurrent lesion, and metastatic disease could not be performed. Third, historical treatment documentation was incomplete. In particular, the dose, fractionation, and duration of the 1977 postoperative radiotherapy were not retrievable, and the exact regimen, dosage, and number of cycles of the 2018 concurrent chemotherapy could not be fully retrieved. Finally, although molecular profiling of the available 2018 recurrent soft palate lesion revealed NOTCH2 copy number gain and structural variants involving SPEN and MTOR, the absence of longitudinal tissue comparison and functional validation prevents us from determining whether the 2018 recurrent lesion and subsequent metastatic lesions represent stepwise clonal evolution from the original primary tumor or biologically distinct later events. Therefore, in this single-case report, these molecular findings should be interpreted as exploratory and hypothesis-generating (12–14).

## Conclusion

TACC is an extremely rare malignancy characterized by prolonged clinical persistence and a high risk of delayed recurrence and distant metastasis. This case demonstrates that long-term survival does not preclude subsequent aggressive progression, even after satisfactory local control. Conventional surgery and radiotherapy may be insufficient to prevent late-stage disease acceleration. Therefore, extended, potentially lifelong surveillance should be considered essential in patients with TACC.

## Data Availability

The datasets presented in this study can be found in online repositories. The names of the repository/repositories and accession number(s) can be found in the article/**Supplementary Material**.

## Supplementary material

Supplementary Table 1 Pathological findings of the patient’s surgical specimens (2018–2022).

Supplementary Table 2 Copy Number Variation (CNV) Profile of the Metastatic Lesion in Soft Palate Adenoid Cystic Carcinoma (ACC).

Supplementary Table 3 Structural Variant (SV) Profile of the recurrent soft palate lesion in Adenoid Cystic Carcinoma (ACC).

Supplementary Table 4 Summary of the PubMed search strategy for ACC case reports in tonsil, soft palate, oropharynx, and minor salivary glands.

Supplementary Figure 1 Radiological findings of the 2018 recurrent soft palate lesion. **(A)** Coronal CT image showing a soft tissue lesion in the oropharyngeal region without definite adjacent bony destruction. **(B)** Contrast-enhanced axial T1-weighted MRI showing a heterogeneously enhancing soft tissue lesion in the left tonsillar fossa/soft palate region, extending anteriorly with a partially indistinct interface with the adjacent soft palate. No definite adjacent bony invasion is observed.

Supplementary Figure 2 Radiological findings of regional cervical nodal disease and suspected locoregional extension. **(A)** Imaging/ultrasound examination in 2020 showing multiple cervical lymph nodes in the submental and submandibular regions bilaterally, with more prominent findings on the right side, clinically suspected to represent regional cervical nodal metastasis. **(B)** Contrast-enhanced MRI showing bilateral skull base meningeal thickening and enhancement, with abnormal soft tissue signal/enhancement in the left cavernous sinus region. **(C)** Contrast-enhanced MRI showing abnormal enhancement of the soft tissues surrounding the bilateral sphenoid sinuses. **(D)** Contrast-enhanced MRI showing abnormal enhancement of the soft tissues adjacent to the left upper alveolar region.

Supplementary Figure 3 Serial chest CT images showing radiological stability of the pulmonary nodule during follow-up. Serial chest CT images obtained at the same anatomical level demonstrate a pulmonary nodule without definite radiological progression during follow-up. **(A)** January 27, 2021. **(B)** July 24, 2022. **(C)** July 28, 2022. **(D)** February 21, 2023.

Supplementary Figure 4 PRISMA flow diagram of literature search and case selection for tonsillar adenoid cystic carcinoma. Flow diagram illustrating the literature search strategy, screening process, eligibility assessment, and final inclusion of reported cases of tonsillar and oropharyngeal adenoid cystic carcinoma.
