# Supplementary material for: Case Report: A rare case of over 45 years’ survival in a patient with tonsillar adenoid cystic carcinoma
Source: Front Oncol. 2026 Jun 2;16:1824507. doi: 10.3389/fonc.2026.1824507 (PMC13268913; doi:10.3389/fonc.2026.1824507)
Supplement: Supplementary Table 1 — Pathological findings of the patient’s surgical specimens (2018–2022). [file Table1.docx]

**Supplementary Table 1. Pathological Findings of the Patient’s Surgical Specimens (2018–2022)**

1. **2018 local recurrence: diagnostic excision and definitive resection**

| **Specimen / admission** | **Procedure** | **Diagnostic purpose** | **Pathological findings** | **Interpretation in this manuscript** |
| --- | --- | --- | --- | --- |
| First 2018 admission | Initial diagnostic local excision of the reconstructed left soft palate lesion | To distinguish post-treatment inflammatory change/fibrosis from tumour recurrence | Intraoperative frozen-section assessment and H&E review of the limited diagnostic tissue showed focal carcinoma infiltration suspicious for recurrent ACC in the clinical context. Permanent sections of the additionally submitted local excision/margin tissue showed chronic inflammatory changes without definite residual carcinoma (IHC results are listed below). Immunohistochemistry listed below was performed on the additionally submitted tissue and was not used as the sole basis for definitive tumour classification.   \| *Marker* \| *Result* \| \| --- \| --- \| \|  \|  \| \| *CK* \| *Positive* \| \| *CK7* \| *Negative* \| \| *CD117* \| *Negative* \| \| *S-100* \| *Partially positive* \| \| *Calponin* \| *Negative* \| \| *Ki-67* \| *~3–5%* \|   *The immunohistochemical results listed for the first 2018 admission refer to the additionally submitted inflammatory/margin tissue, not to the focal carcinoma infiltration identified in the limited diagnostic tissue. | Raised suspicion for recurrent carcinoma but was not used as the sole basis for definitive tumour classification |
| Second 2018 admission | Radical resection of the left soft palate lesion via mandibulotomy | Definitive resection and pathological classification | Recurrent ACC of the left soft palate, approximately 2.0 × 2.0 × 0.8 cm, with infiltrative growth and focal perineural invasion. Tumour was present at the cauterized resection surface, suggesting a close/involved margin; exact margin distances and depth of invasion were not documented. Lymphovascular invasion could not be definitively assessed based on the available pathological material. (IHC results are listed below)   \| *Marker* \| *Result* \| \| --- \| --- \| \| *CK8/18* \| *Positive* \| \| *CK7* \| *Positive* \| \| *CD117* \| *Positive* \| \| *p63* \| *Partially positive* \| \| *Calponin* \| *Negative* \| \| *Ki-67* \| *~30%* \| | Established the final diagnosis of recurrent ACC |

1. **Additional SCC-related immunohistochemistry**

| **Specimen** | **Marker** | **Result** | **Interpretation** |
| --- | --- | --- | --- |
| 2018 radical resection specimen | p40 | *Negative* | No diffuse sheet-like squamous marker expression |
|  | CK5/6 | *Partially positive* | No diffuse sheet-like squamous marker expression |

1. **2022 metastatic cervical lymph node specimens**

| **Specimen** | **Procedure** | **Histopathological findings** | **Number of lymph nodes examined** | **Number of positive nodes** | **Immunohistochemistry** | **Ki-67 index** |
| --- | --- | --- | --- | --- | --- | --- |
| **Right level II lymph node** | Ultrasound-guided FNA / biopsy | Metastatic ACC, predominantly cribriform 80% with tubular component 20% | – | – | CD117+, CK7+, partial GFAP+, partial calponin+, DOG-1− | ~10% |
| **Left level I lymph nodes** | Neck dissection | Metastatic adenoid cystic carcinoma | 4 | 1 | Not assessed | Not available |
| **Left level IIa lymph nodes** | Neck dissection | No metastatic tumour identified | 3 | 0 | Not assessed | Not available |
| **Left level IIb lymph nodes** | Neck dissection | Fibrofatty tissue | – | 0 | Not assessed | Not available |
| **Left level III lymph nodes** | Neck dissection | Fibrofatty tissue with minor salivary tissue | – | 0 | Not assessed | Not available |
| **Right level I lymph nodes** | Neck dissection | Metastatic adenoid cystic carcinoma | 4 | 1 | Not assessed | Not available |
| **Right level IIb lymph nodes** | Neck dissection | No metastatic tumour identified | 17 | 0 | Not assessed | Not available |
| **Right level III lymph nodes** | Neck dissection | Metastatic adenoid cystic carcinoma | 16 | 1 | Not assessed | Not available |
| **Left upper alveolar gingival lesion** | Biopsy | Chronic inflammatory mucosa, no tumour identified | – | – | CK+, IgG+, IgG4– | ~3–5% |

ACC, adenoid cystic carcinoma.

“–” denotes data not available or not applicable.

Ki-67 proliferation index was evaluated only in specimens with adequate viable tumour tissue.

Immunohistochemical analysis was not routinely performed on all lymph node specimens.
