# Supplementary material for: Case Report: A rare case of over 45 years’ survival in a patient with tonsillar adenoid cystic carcinoma
Source: Front Oncol. 2026 Jun 2;16:1824507. doi: 10.3389/fonc.2026.1824507 (PMC13268913; doi:10.3389/fonc.2026.1824507)
Supplement: Supplementary Table 2 — Copy Number Variation (CNV) Profile of the Metastatic Lesion in Soft Palate Adenoid Cystic Carcinoma (ACC). [file Table2.docx]

**Supplement table 2.Copy Number Variation (CNV) Profile of the Metastatic Lesion in Soft Palate Adenoid Cystic Carcinoma (ACC).**

| gene | chr | start | end | log2 | depth | cn |
| --- | --- | --- | --- | --- | --- | --- |
| NOTCH2: exon21 | chr1 | 120479904 | 120480089 | 1.18588 | 218.897 | 5 |
| NOTCH2: exon20 | chr1 | 120480479 | 120480633 | 1.18588 | 270.396 | 5 |
| NOTCH2: exon19 | chr1 | 120483177 | 120483379 | 1.18588 | 276.911 | 5 |
| NOTCH2: exon18 | chr1 | 120484148 | 120484377 | 1.18588 | 284.24 | 5 |
| NOTCH2: exon17 | chr1 | 120491036 | 120491189 | 1.18588 | 300.902 | 5 |
| NOTCH2: exon16 | chr1 | 120491588 | 120491812 | 1.18588 | 339.607 | 5 |
| NOTCH2: exon15 | chr1 | 120493328 | 120493478 | 1.18588 | 274.607 | 5 |
| NOTCH2: exon14 | chr1 | 120496164 | 120496389 | 1.18588 | 297.48 | 5 |
| NOTCH2: exon13 | chr1 | 120497662 | 120497855 | 1.18588 | 336.347 | 5 |
| NOTCH2: exon12 | chr1 | 120501995 | 120502145 | 1.18588 | 420.487 | 5 |

Gene: Gene name and genomic location

Chr: Chromosome number

Start: Start position of the genomic region

End: End position of the genomic region

Log2: Calculated Log2 ratio value for the region

Depth: Average sequencing coverage depth of the region

CN: Copy number of the region
