# Supplementary material for: Case Report: A rare case of over 45 years’ survival in a patient with tonsillar adenoid cystic carcinoma
Source: Front Oncol. 2026 Jun 2;16:1824507. doi: 10.3389/fonc.2026.1824507 (PMC13268913; doi:10.3389/fonc.2026.1824507)
Supplement: Supplementary Table 3 — Structural Variant (SV) Profile of the recurrent soft palate lesion in Adenoid Cystic Carcinoma (ACC). [file Table3.docx]

Supplementary Table 3. Structural Variant (SV) Profile of the Metastatic Lesion in Soft Palate Adenoid Cystic Carcinoma (ACC).

| chr_start | start | chr_end | end | sv_type | ratio | start_gene | end_gene |
| --- | --- | --- | --- | --- | --- | --- | --- |
| chr1 | 11167562 | chr1 | 11167785 | INV | - | MTOR | MTOR |
| chr1 | 11291157 | chr1 | 242168749 | DEL | - | MTOR | - |
| chr1 | 16160780 | chr1 | 16237703 | DEL | - | FLJ37453 | SPEN |
| chr1 | 16255294 | chr1 | 16258199 | INV | - | SPEN | SPEN |
| chr1 | 16259431 | chr1 | 16260025 | DUP | - | SPEN | SPEN |
| chr1 | 16260606 | chrM | 12386 | BND | - | SPEN | - |
| chr1 | 16261288 | chr1 | 16261671 | DUP | - | SPEN | SPEN |
| chr1 | 16262688 | chr1 | 21815774 | INV | - | SPEN | - |
| chr1 | 16265275 | chr1 | 16265532 | INV | - | SPEN | SPEN |
| chr1 | 87311254 | chr1 | 244676131 | DEL | - | - | CATSPERE |

chr_start: Chromosome number of the start position of the left gene

start: Start position of the left gene

chr_end: Chromosome number of the start position of the right gene

end: Start position of the right gene

sv_type: SV type: DEL (large fragment deletion), INS (large fragment insertion), DUP (tandem duplication), INV (chromosome inversion), BND (chromosome translocation)

ratio: Fusion frequency

start_gene: Name of the left gene

end_gene: Name of the right gene
