# Supplementary material for: Case Report: A rare case of over 45 years’ survival in a patient with tonsillar adenoid cystic carcinoma
Source: Front Oncol. 2026 Jun 2;16:1824507. doi: 10.3389/fonc.2026.1824507 (PMC13268913; doi:10.3389/fonc.2026.1824507)
Supplement: Supplementary Table 4 — Summary of the PubMed search strategy for ACC case reports in tonsil, soft palate, oropharynx, and minor salivary glands. [file Table4.docx]

**Supplementary Table 4.** Summary of the PubMed search strategy for ACC case reports in tonsil, soft palate, oropharynx, and minor salivary glands

| Item | Details |
| --- | --- |
| **Database** | PubMed |
| **Search Period** | From inception to December 2025 |
| **Example Search String** | ("adenoid cystic carcinoma" OR ACC) AND (tonsil OR "soft palate" OR oropharynx OR "minor salivary gland") AND (recurrence OR metastasis OR "long-term follow-up") AND ("case reports"[Publication Type]) |
| **Study Type** | Case reports |
| **Inclusion Criteria** | Related to adenoid cystic carcinoma (ACC); located in tonsil, soft palate, oropharynx, or minor salivary glands; reporting recurrence, metastasis, or long-term follow-up |
| **Exclusion Criteria** | Not in the above anatomical sites or not relevant to ACC; not case report type |
