# Supplementary figures and images for: Case Report: A rare case of over 45 years’ survival in a patient with tonsillar adenoid cystic carcinoma
Source: Front Oncol. 2026 Jun 2;16:1824507. doi: 10.3389/fonc.2026.1824507 (PMC13268913; doi:10.3389/fonc.2026.1824507)

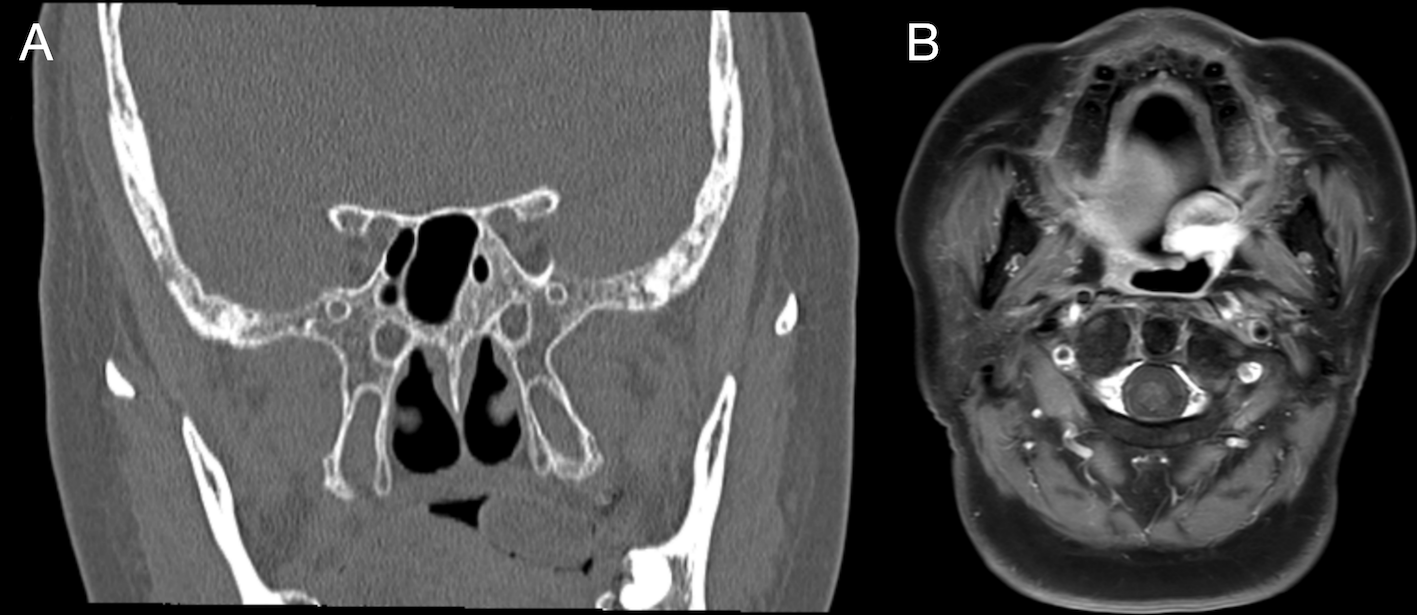

Supplement: Supplementary Figure 1 — Radiological findings of the 2018 recurrent soft palate lesion. (A) Coronal CT image showing a soft tissue lesion in the oropharyngeal region without definite adjacent bony destruction. (B) Contrast-enhanced axial T1-weighted MRI showing a heterogeneously enhancing soft tissue lesion in the left tonsillar fossa/soft palate region, extending anteriorly with a partially indistinct interface with the adjacent soft palate. No definite adjacent bony invasion is observed. [file Image1.jpeg]

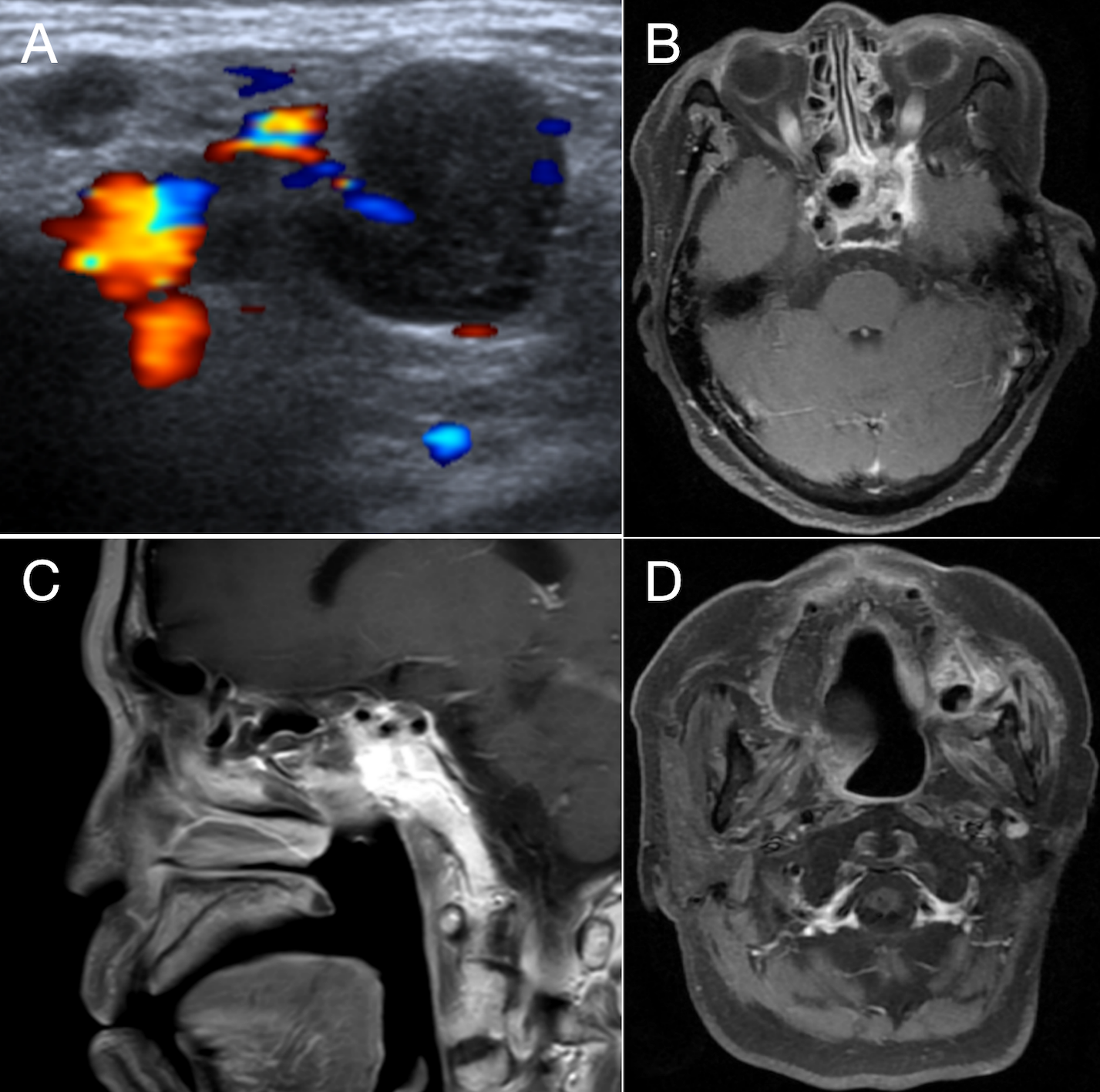

Supplement: Supplementary Figure 2 — Radiological findings of regional cervical nodal disease and suspected locoregional extension. (A) Imaging/ultrasound examination in 2020 showing multiple cervical lymph nodes in the submental and submandibular regions bilaterally, with more prominent findings on the right side, clinically suspected to represent regional cervical nodal metastasis. (B) Contrast-enhanced MRI showing bilateral skull base meningeal thickening and enhancement, with abnormal soft tissue signal/enhancement in the left cavernous sinus region. (C) Contrast-enhanced MRI showing abnormal enhancement of the soft tissues surrounding the bilateral sphenoid sinuses. (D) Contrast-enhanced MRI showing abnormal enhancement of the soft tissues adjacent to the left upper alveolar region. [file Image2.jpeg]

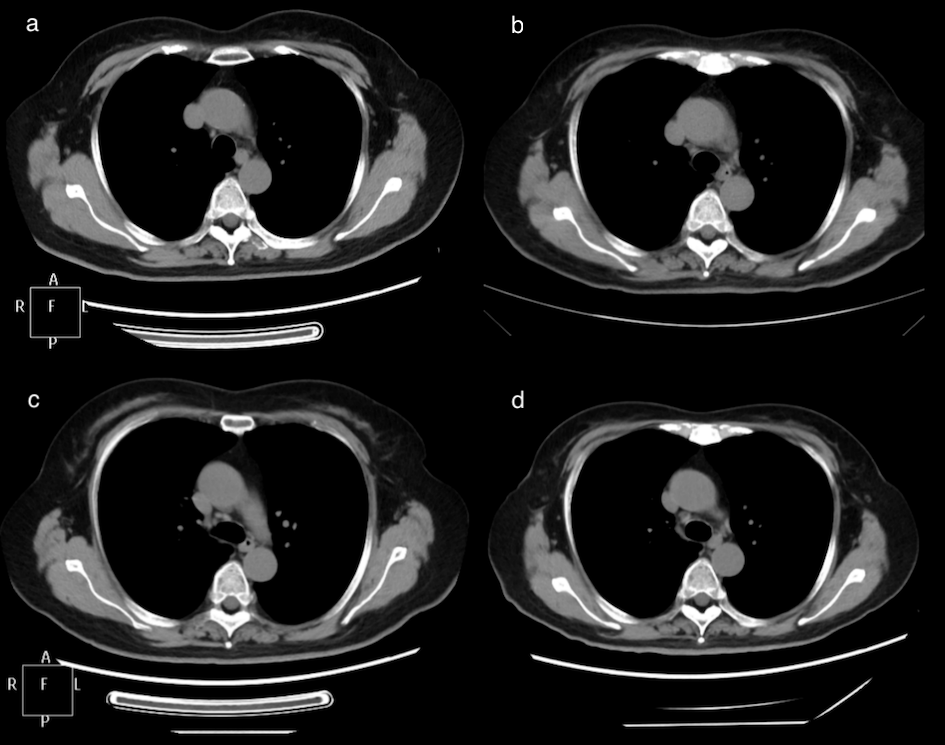

Supplement: Supplementary Figure 3 — Serial chest CT images showing radiological stability of the pulmonary nodule during follow-up. Serial chest CT images obtained at the same anatomical level demonstrate a pulmonary nodule without definite radiological progression during follow-up. (A) January 27, 2021. (B) July 24, 2022. (C) July 28, 2022. (D) February 21, 2023. [file Image3.jpeg]

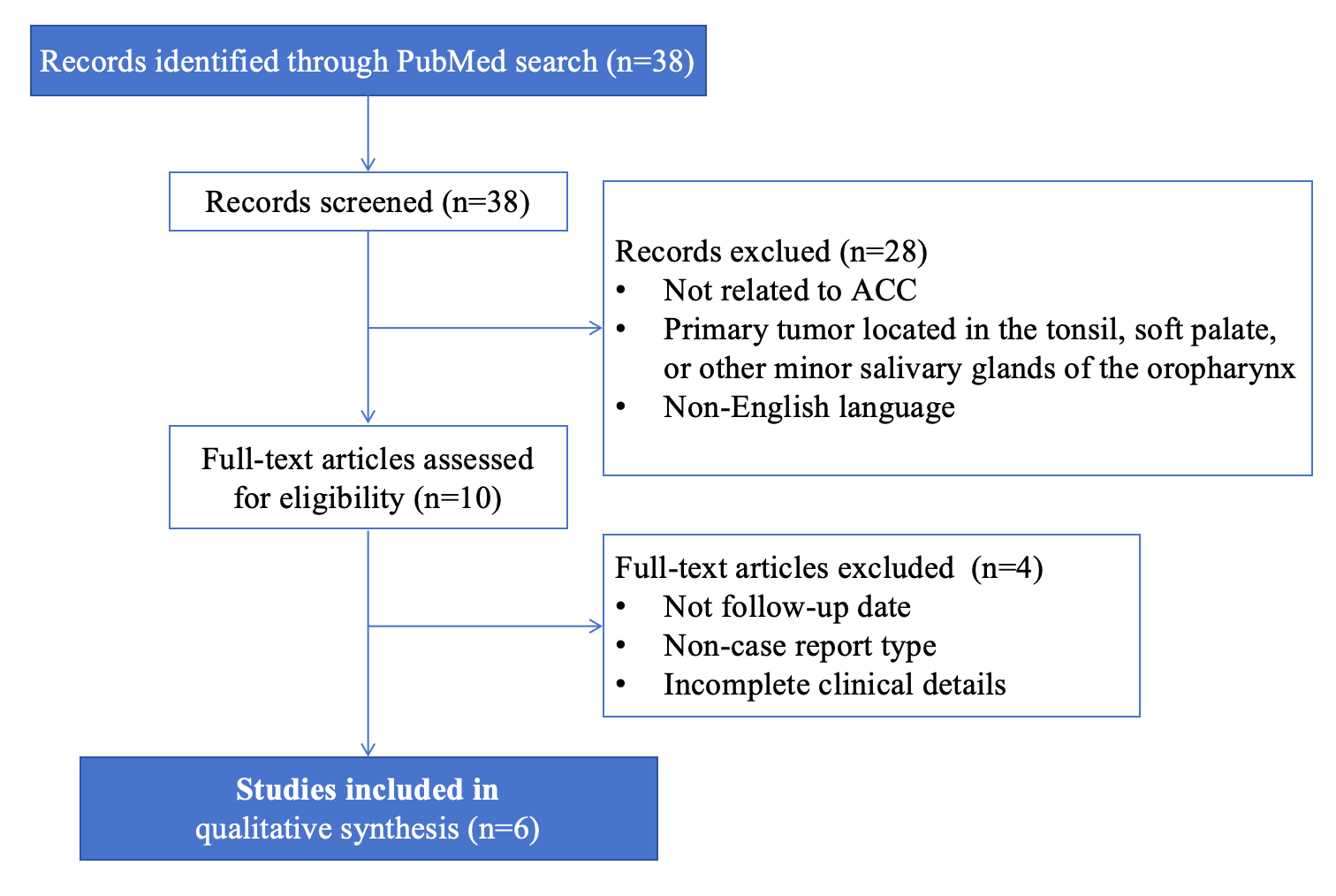

Supplement: Supplementary Figure 4 — PRISMA flow diagram of literature search and case selection for tonsillar adenoid cystic carcinoma. Flow diagram illustrating the literature search strategy, screening process, eligibility assessment, and final inclusion of reported cases of tonsillar and oropharyngeal adenoid cystic carcinoma. [file Image4.jpeg]
